# Impact of Timing During Simulated Vitreoretinal Surgery by Trainees

**DOI:** 10.7759/cureus.88635

**Published:** 2025-07-23

**Authors:** Ashish Markan, Basavaraj Tigari, Simar Rajan Singh, Deeksha Katoch, Mohit Dogra, Reema Bansal, Vishali Gupta, Ramandeep Singh

**Affiliations:** 1 Ophthalmology, All India Institute of Medical Sciences, New Delhi, Delhi, IND; 2 Ophthalmology, Postgraduate Institute of Medical Education and Research, Chandigarh, IND; 3 Advanced Eye Center, Postgraduate Institute of Medical Education and Research, Chandigarh, IND

**Keywords:** eyesi surgical, fatigue, simulator training, surgical outcome, timing of surgery, trainees, vitreoretinal surgery

## Abstract

Purpose: This study aimed to examine the impact of surgical timing on the performance of vitreoretinal (VR) trainees in a simulated setting.

Methods: In a prospective cross-over observational study, VR trainees were asked to perform various surgical tasks on the Eyesi surgical simulator. These included navigation and anti-tremor, pars plana vitrectomy and posterior vitreous detachment, bimanual training, bimanual scissors use, and epiretinal membrane peeling. The trainees were randomly assigned to Group A (performed tasks during morning hours) and Group B (performed tasks during evening hours). After completing assigned tasks, the trainees were asked to cross over to the other group. Primary outcome measures were objective scores and time to perform each surgical maneuver in both groups. Secondary outcome measures included subjective scores given by each trainee and complications encountered.

Results: Eight VR trainees, three males (37.5%) and five females (62.5%), were included in the study. The mean objective score obtained after performing various surgical tasks was similar in both groups. The time taken to complete the above tasks was identical. Though the complications were more in the evening than in the morning, the difference was not statistically significant. Subjectively, residents did not find any difference in either group.

Conclusions: The timing of VR surgery does not alter the surgical performance in a simulator setting. While objective measures showed no significant difference, complications trended higher in the evening.

## Introduction

Prolonged work hours and insufficient recovery are known to affect the homeostatic and circadian processes, leading to fatigue in the body [1,2]. Fatigue refers to exhaustion from exertion, labour and stress [3]. The Institute of Medicine (IOM) report in 1999 showed medical errors to be the most common cause of adverse medical events [4]. Furthermore, the report highlighted that physician fatigue was the primary cause of these medical errors. Since then, efforts have been made to regulate resident work hours to prevent fatigue-related complications and improve patient safety [1,5]. Resident trainees are often subjected to long working hours [6]. Sleep deprivation, fatigue and prolonged working hours have been associated with medical errors and increased attention failures by the interns [7,8]. The effect of fatigue on the surgical performance of residents needs to be clearly understood.

Several real-world and simulator-based studies have reported conflicting results [8-10]. Some studies have demonstrated that muscular and mental fatigue negatively impact the surgeon’s fine motor control and instrument handling [11-13]. By contrast, other studies found similar complication rates and no significant difference in surgical performance amongst fatigued and rested surgeons [3,14]. 

Studies have utilised simulators to examine the effect of fatigue on a surgeon's performance without jeopardising the patient’s safety [9, 15]. Virtual reality simulators have been in use in the aviation industry to train pilots and objectively assess their performance [16]. Simulators provide a real-time surgical experience to beginners, allowing them to learn skills and objectively evaluate their performance without harming the patients [17].

In this study, we prospectively evaluated the impact of surgery timing on vitreoretinal (VR) surgical performance using a simulator-based model.

## Materials and methods

This was a prospective cross-over observational study. VR trainees performed various surgical tasks using the posterior segment module of the Eyesi Surgical simulator, version 3.4.2 (VR Magic Holding AG, Germany). The study was conducted as per tenets of the Declaration of Helsinki. Ethical clearance was obtained from the Institutional Review Board (IRB) of Postgraduate Institute of Medical Education and Research (PGIMER), Chandigarh (NK/7711/Study/294, dated 14-9-2021). The study was conducted over a period of three months from December 2021 to February 2022. No financial incentives were offered to the trainees to be part of the study. Informed participation consent was taken from all the trainees.

**Figure 1:**
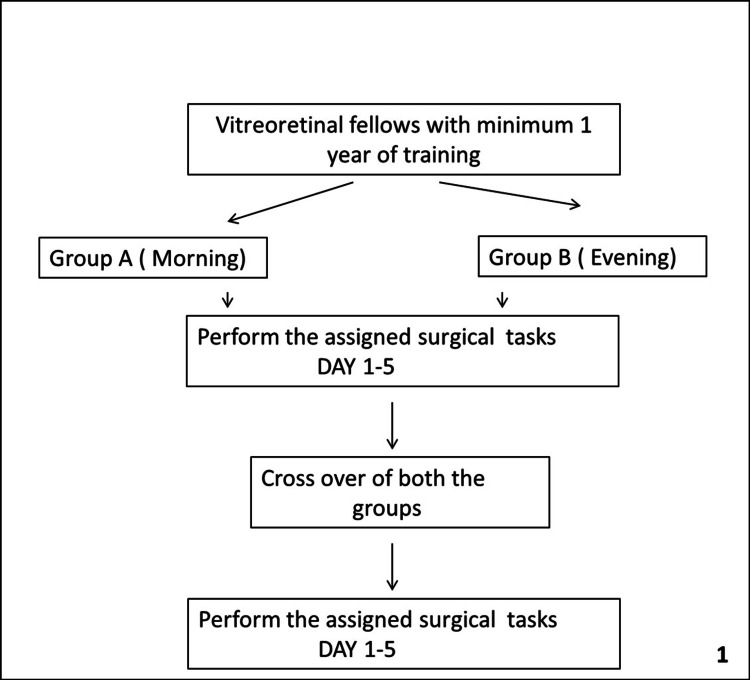
Flowchart showing the methodology of simulated surgeries performed by vitreoretinal trainees.

All VR trainees with at least one year of VR surgical training were included in the study. Trainees with less than one year of experience were excluded from the study. The trainees were randomised into two groups, as per Figure 1.

The trainees were asked to perform the following tasks each day: navigation and anti-tremor (level 3), pars plana vitrectomy (PPV) and posterior vitreous detachment (PVD) (level 6), bimanual training (level 3), bimanual scissors (level 3) and epiretinal membrane (ERM) peeling (level 4). The levels were chosen to achieve an intermediate level of difficulty and mimic the real-world scenario, ensuring that all the trainees could perform the tasks. The trainees were given one hour the day prior to familiarise themselves with the simulator before performing the final task. They were instructed to complete each task three times, and then an average score was recorded.

In this study, we established that any score greater than 0 was sufficient for the user to go to the next step. Each trainee was instructed to perform one task each day. This allowed four trainees to perform tasks in a single day. The Eyesi machine objectively graded the score between 0 and 100 for each task. Various studies have used the objective scoring given by the Eyesi machine to study the factors that affect surgical performance [3,14].

After completing the task, the trainees in the morning group were asked to perform the same tasks in the evening and vice versa. This allowed a crossover between the two groups and thus eliminating any selection bias. For Group A, it was ensured that all trainees had at least six hours of sleep. This ensured that trainees in Group A had adequate rest before performing the surgical manoeuvres. As for trainees in Group B, they performed the surgical tasks after eight to nine hours of a continuous duty shift and were assumed to be fatigued.

Primary outcome measures included objective scores and time to perform each surgical task, given at the end of the task. Secondary outcome measures included subjective scores given by each trainee and intraoperative complications encountered. The intraoperative complications were recorded as the total number of complications encountered during three attempts on the simulator. Subjective experience was graded on a 1-5 scale, with 1 = low, 2 = below average, 3 = average, 4 = good and 5 = excellent surgical experience.

Statistical tests were conducted using IBM SPSS Statistics for Windows version 29 (IBM Corp., Armonk, NY). Categorical variables were measured as percentages, and continuous variables were calculated as means. Tests of normality were applied to determine the distribution of data. An unpaired t-test was used for normally distributed data, and Wilcoxon signed-rank test was used for skewed data. A p-value < 0.05 was considered statistically significant.

This study was designed as a retrospective pilot analysis to explore clinical trends and outcomes in a specific subset of patients. As such, a formal power calculation was not performed a priori.

## Results

A total of eight VR trainees, three males (37.5%) and five females (62.5%), were included in the study. The mean age of the male and female trainees was 34±4.58 years and 30.8±4.08 years, respectively.

**Figure 2:**
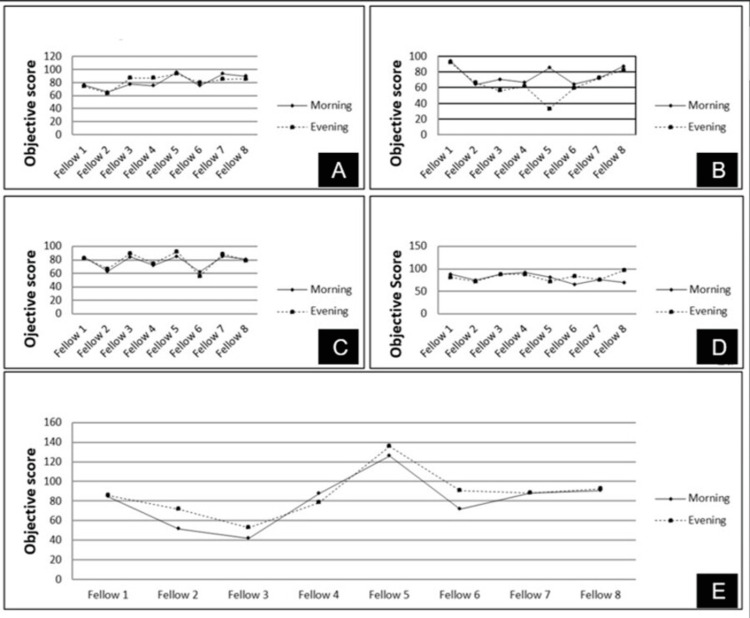
Line diagrams showing objective scores obtained by each fellow while performing various surgical tasks (navigation and anti-tremor (2a), pars plana vitrectomy (PPV) and posterior vitreous detachment (PVD) induction (2b), bimanual training (2c), bimanual scissors (2d) and ERM peeling (2e)) during morning and evening hours.

**Figure 3:**
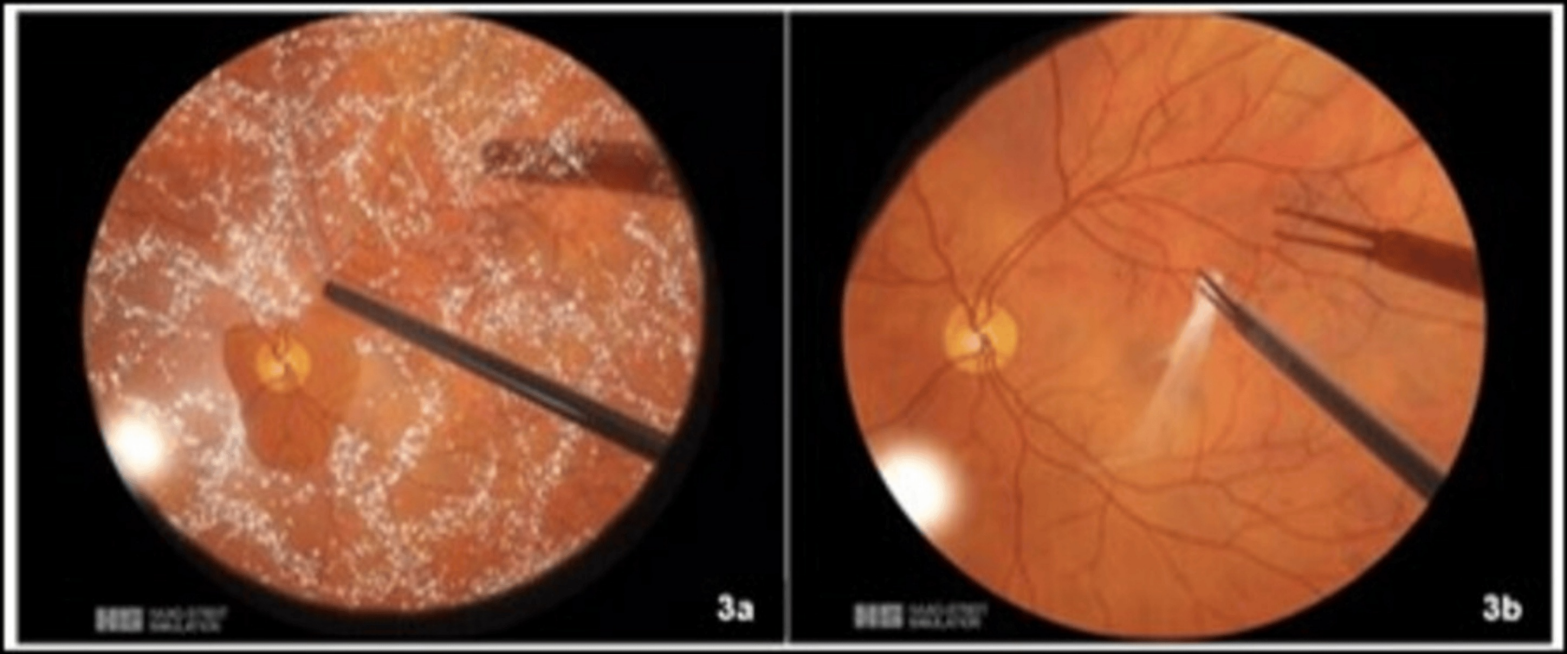
Intraoperative snapshots of the vitreoretinal (VR) surgical simulator demonstrating pars plana vitrectomy (3a) and epiretinal membrane (ERM) peeling (3b).

The mean objective score obtained during morning and evening shifts to perform various tasks was as follows: navigation and anti-tremor (81.37±10.66 and 82.42±9.35, respectively, p-value: 0.68, Figure 2a), PPV and PVD (76.02±11.7 and 65.99±17.81, respectively, p-value: 0.154, Figure 2b), bimanual training (77.23±9.88 and 79.02±12.37, respectively, p-value: 0.267, Figure 2c), bimanual scissors (79.82±9.46 and 83.16±8.84, respectively, p-value: 0.487, Figure 2d) and ERM peeling (80.44±25.85 and 87.28±23.54, respectively, p-value: 0.094, Figure 2e) (Table 1, Figures 2, 3).

**Table 1:** Comparison of objective scores, subjective scores and time taken to perform various surgical tasks in morning and evening time. PPV: pars plana vitrectomy, PVD: posterior vitreous detachment, ERM: epiretinal membrane

	Objective score (0-100)			Subjective score (1-5)			Time taken (sec)		
	Morning	Evening	P-value (95% CI)	Morning	Evening	P-value (95% CI)	Morning	Evening	P-value (95% CI)
Navigation/anti-tremor	81.37±10.66	82.42±9.35	0.68 (-11.8, 9.7)	3.85±0.37	3.71±0.48	0.35 (-0.32, 0.6)	166±127.9	190±167.72	0.61 (-183.9, 135.94)
PVD/PPV	76.02±11.7	65.99±17.81	0.15 (-6.13, 26.19)	4.0±0.57	3.71±0.75	0.45 (-0.42, 1)	369.38±238	411±240.7	0.23 (-298.3, 215.06)
Bimanual training	77.23±9.88	79.02±12.37	0.26 (-13.79, 10.21)	4.28±0.75	4.42±0.78	0.35 (-0.96, 0.68)	93.42±45.24	115.29±88.59	0.41 (-97.3, 53.56)
Bimanual scissors	79.82±9.46	83.16±8.84	0.48 (-13.16, 6.48)	4.14±0.69	4.28±0.75	0.35 (-0.91, 0.63)	76.95±25.05	80.12±34.2	0.53 (-35.32, 28.98)
ERM peeling	80.44±25.85	87.28±23.54	0.09 (-33.35, 19.67)	3.28±1.38	3.14±1.57	0.60 (-1.45, 1.73)	342.21±141.28	381.79±133.95	0.24 (-187.2, 108.05)

The VR trainees provided a subjective score as per their experience while performing various tasks on the simulator. The mean subjective score to perform multiple functions during morning and evening slots was similar (Table 1).

The total time (seconds) taken during morning and evening is shown in Table 1. Although the time taken in the evening was slightly more than morning, the difference did not reach any significant value (p-value > 0.05).

Iatrogenic retinal breaks were the most common intraoperative complications during the morning and evening sessions (Table 2).

**Table 2:** Complications encountered during the performance of various tasks by the vitreoretinal (VR) trainees PPV: pars plana vitrectomy, PVD: posterior vitreous detachment, ERM: epiretinal membrane

Surgical task	Morning	Evening
Navigation/anti-tremor	None	Working outside microscope (n=1, 12.5%)
PVD/PPV	Retinal break (n=3, 37.5%), stress injury to peripheral retina (n=1, 12.5%)	Retinal break (n=4, 50%), stress injury to peripheral retina (n=1, 12.5%), lens touch (n=1, 12.5%)
Bimanual training	Reduced stability (n=1, 12.5%)	Retinal tears (n=2, 25%), reduced stability (n=1, 12.5%)
Bimanual scissors	Injury to retina (n=1, 12.5%)	None
ERM peeling	Retinal break (n=2, 25%), bleed (n=1, 12.5%), removing open forceps (n=1, 12.5%)	Retinal break (n=3, 37.5%), retinal injury (n=1,12.5%), bleed (n=1, 12.5%)
Total events of complications	10	15
Frequency of retinal breaks	5	9

The frequency of intraoperative complications was higher in the evening than in the morning, but it was not statistically significant (p = 0.125). Other reported complications were stress injury to the retina, lens touch, working outside the microscope, reduced stability, and retinal bleed.

## Discussion

The results of our study show that surgery timings do not affect the performance to perform tasks given to VR trainees in a simulated setting. This, in turn, suggests that fatigue does not affect the surgical performance of VR trainees on the surgical simulator.

Patient safety is a growing concern in modern-day medical practice. Providing optimum outcomes to the patient is of utmost importance [18]. Several non-technical factors can act as stressors, especially in novice surgeons. [19] However, there is no substantial evidence that these factors affect the surgical performance [19,20]. Non-technical skills have been strongly emphasised in surgical training by the New Intercollegiate Curriculum for Surgical Education, London, UK [21]. It is essential to gauge the residents’ surgical skills and assess their performance in different real-world clinical scenarios.

The effect of fatigue on surgical performance has been widely studied in real-world and simulator-based surgeries [1]. Most studies have looked at the impact of fatigue on non-ophthalmic surgeries, especially on surgical simulators, whose objective surrogates correlate with actual world outcomes [3,9]. The effect of fatigue on surgical outcomes is variable, and no consolidated evidence exists that fatigue hampers surgical performance.

The effect of fatigue on ophthalmic surgeries is rarely reported and is limited to anterior segment surgeries [3,14]. Our study showed that the timing of performing VR surgery did not alter the surgical results under a simulated environment. Our results are consistent with a study by Waqar et al. [3]. The authors showed that fatigue had no detrimental effects on surgical performance. Our study differed in a few aspects from Waqar et al. Firstly, our study aimed at performing surgical steps involved in posterior segment surgeries. They focused on the level four forceps module used in cataract surgeries. Secondly, our study involved trainees and not experienced surgeons, as in the study by Waqar et al. [3]. Both studies highlight that fatigue is unlikely to hamper the surgical performance of both anterior and posterior segment surgeons, whether trainees or experienced surgeons.

Erie et al. have also shown that there is no effect of sleep deprivation on the performance of ophthalmology residents, whether the surgical tasks were performed in a rested state, after an eight-hour workday or in a fatigued individual [14]. The surgical tasks were limited to the forceps and anti-tremor modules, unlike our study, where seven different surgical tasks routinely performed in VR surgeries were studied. In addition, there was no difference in time taken to complete the tasks amongst rested, fatigued and sleep-deprived residents in a study by Erie et al. This is consistent with the results of our research. Contrary to this, Yamany et al. showed that fatigue hampered surgical skills and increased the time to complete suturing in urology and general surgery residents [22].

Complications encountered during the fatigued state were higher than in the rested state, although this difference was not statistically significant. Similarly, the lens injury score was higher in the fatigued state than the rested state in a study by Waqar et al. [3], although the values did not reach significance. This could be attributed to a smaller sample size and warrants further investigation with a larger sample size.

Our fellows did not feel any subjective difference while performing during different times of the day. This could be because young surgeons have a high motivation to learn and a better attention span. This helps them stay focused despite being tired while performing a skilled task. Schlosser et al. have shown call-associated fatigue to be a predominantly subjective perception [23]. There is also the notion that surgeons self-select themselves into surgical specialities because they believe they can perform even when tired. Conversely, those who feel incapable of performing under sleep-deprived and fatigued conditions enter into non-surgical specialities [24]. Studies have shown that fatigue hampers cognitive skills more than psychomotor skills [11]. Performing surgical tasks on the simulator is predominantly a psychomotor skill. This could explain no effect of fatigue in our study and previous studies [3,14,25].

Apart from fatigue, several other factors like alcohol intake, caffeine intake, poor assistance, distractions in OT and emotional stress can negatively influence the surgeon’s performance [1,26]. Caffeine intake is associated with poor surgical performance owing to decreased dexterity and increased tremors. Contrary to this, propranolol and other beta-blockers have been shown to attenuate these tremors and improve surgical performance [27].

Our study has a few limitations. Firstly, we did not consider the influence of the above external factors, which could have affected surgical performance. Secondly, the study had a limited sample size. The sample size of eight patients represents all eligible cases managed during the study period that met the inclusion criteria. While the study is not powered to detect small effect sizes, it aims to generate preliminary data that can inform the design and sample size of future prospective studies. Thirdly, no published evidence regarding the Eyesi simulator supports the transfer of skills from the simulator to the operating room. As a result, it is difficult to comment on the effect of fatigue on real-world surgical performance. Lastly, as some of the trainees already had some surgical experience, they might be accustomed to the usual exhaustion of the day and could perform better. The main strength of this study is that it is, to the best of our knowledge, the first to evaluate the impact of surgical timing and fatigue on the performance of VR trainees in simulated conditions.

## Conclusions

The timing of performing vitreoretinal surgery does not alter surgical performance in a simulated setting. This can be attributed to the high motivation to learn and a good attention span of trainees at a young age. Our findings suggest that psychomotor skills involved in surgical performance may be resilient to the effects of fatigue, at least in the short term. This supports the continued use of surgical simulators in training environments without the concern of time-of-day bias. However, further studies with larger cohorts and real-world surgical assessments are needed to validate these results and explore the broader implications on patient outcomes.
